# Parasite Burden in Hamsters Infected with Two Different Strains of *Leishmania* (*Leishmania*) *infantum*: “Leishman Donovan Units” versus Real-Time PCR

**DOI:** 10.1371/journal.pone.0047907

**Published:** 2012-10-24

**Authors:** Nádia das Dores Moreira, Juliana Vitoriano-Souza, Bruno Mendes Roatt, Paula Melo de Abreu Vieira, Henrique Gama Ker, Jamille Mirelle de Oliveira Cardoso, Rodolfo Cordeiro Giunchetti, Cláudia Martins Carneiro, Marta de Lana, Alexandre Barbosa Reis

**Affiliations:** 1 Laboratório de Imunopatologia, Núcleo de Pesquisas em Ciências Biológicas/NUPEB, Universidade Federal de Ouro Preto, Ouro Preto, Minas Gerais, Brasil; 2 Laboratório de Pesquisas Clínicas, Departamento de Análises Clínicas, Escola de Farmácia, Universidade Federal de Ouro Preto, Ouro Preto, Minas Gerais, Brasil; 3 Laboratório de Doença de Chagas, Núcleo de Pesquisas em Ciências Biológicas/NUPEB, Departamento de Análises Clínicas, Escola de Farmácia, Universidade Federal de Ouro Preto, Ouro Preto, Minas Gerais, Brasil; Universidade Federal de Minas Gerais, Brazil

## Abstract

To develop and test new therapeutics and immune prophylaxis strategies for visceral leishmaniasis (VL), understanding tissue parasitism evolution after experimental infection with *Leishmania infantum* is important. Experimental infection in a hamster model (*Mesocricetus auratus*) reproduces several typical aspects of canine and human VL that are closely related to the inoculum’s route. We quantified the parasitism in the liver and spleen of hamsters experimentally infected by various routes (intradermal, intraperitoneal, and intracardiac [IC]) and different strains of *L. infantum* (MHOM/BR/74/PP75 and Wild) and compared two different methodologies to evaluate tissue parasitism (Leishman Donovan units [LDU] and real-time qPCR). In addition, the quantification of specific total-IgG in the serum of uninfected and infected hamsters was determined by ELISA. The animals were followed for 1, 3, 6 and 9 months post-infection for survival analysis. We found that infection with the Wild strain by the IC route resulted in higher mortality. Positive antibody (IgG) responses were detected with higher peaks at 6 and 9 months in the IC group inoculated with PP75 strain. However, in animals infected with the Wild strain the IgG levels were elevated in all infected groups during all the time evaluated. We also observed by LDU analysis that the IC route lead to higher parasitism in the liver and spleen with both strains. Furthermore, qPCR showed higher sensitivity for identifying animals with low parasitic burden. In conclusion, qPCR can be useful for assessing parasitism in the spleen and liver of a hamster model infected with *L. infantum* independent of the route of infection, and this technique may become an essential tool for assessing parasite density in the hamster model after experimental treatment or immunization with potential vaccine candidates.

## Introduction

Leishmaniasis is one of the most prevalent neglected tropical diseases affecting public health worldwide. Three hundred fifty million people are considered at risk of contracting leishmaniasis, and some 2 million new cases occur yearly [1]. Visceral leishmaniasis (VL) is characterized in its typical form (kala-azar) by high fever, weight loss, hepatosplenomegaly, and lymphadenopathy. Associated laboratory abnormalities include pancytopenia, leukopenia, hypergammaglobulinemia, and hypoalbuminemia, with intense and widespread parasitism in vital organs such as the liver, spleen, and bone marrow [1], [2], [3].

Under experimental conditions, progression of visceral disease depends on the route of infection as well as the strain of *Leishmania* parasites [4]. Several studies using experimental murine models of VL have been developed, but these inadequately represent human disease. Humans, dogs, and hamsters often exhibit severe clinical signs and symptoms during visceral infection [5], [6], [7], whereas mice generally show few minor clinical signs or none at all, depending mainly on the size of the parasite inoculum [8], [9]. Balb/c mice are typically used to study the visceral disease due to their susceptibility, and *Leishmania donovani* and *L. infantum* infections can be successfully established via intravenous or intradermal administration of the parasite; however, these infections do not reproduce the characteristics of human or canine VL [9].

*Leishmania*-specific experimental infection in a hamster model (*Mesocricetus auratus*) reproduces many aspects of canine and human VL, such as pancytopenia characterized by leukopenia, anemia, cachexia, hypergammaglobulinemia, and absence of T-cell response [10], [11], [12]. The latter dysfunction has been attributed to the inability of antigen-presenting cells to induce specific T cells [13]. Several researchers have considered this dysfunction to be a key element in the progression of VL in the hamster model that could also play a major role in human and canine disease [9], [14], [15]. As a consequence of the absent T-cell response, uncontrolled replication of the parasite occurs, despite strong type-1 cytokine production (interleukin-2, interferon-γ, and tumor necrosis factor [TNF]-α) in the liver, spleen, and bone marrow of animals, leading to a pattern of progressive disease in this model [6].

However, the influence of the inoculum is particularly important in infections in experimental models and various routes have been used. The usual routes of infection in the hamster model of VL are intracardiac and intraperitoneal. Although subcutaneous and intradermal routes are more representative of natural infection, few studies have used these routes [16], [17]. So far, only one study exists in which the authors used the experimental infection through the sandfly bite [18], possibly due to the logistical difficulty in maintaining a *Lutzomyia longipalpis* colony in the laboratory.

The quantification of parasitism in hamsters experimentally infected with *L. infantum* or *L. donovani* is generally determined by classical methods, such as limiting dilution and/or by Leishman Donovan units (LDU) according to Stauber [19]; however, these methods exhibit low sensitivity [20], [21], [22]. The development of a technique that allows quantifying the amastigotes in different parasitized tissues is extremely important to evaluate the impact of parasitism in experimentally infected hamsters. Few studies have evaluated the use of real-time qPCR to quantify the parasite density in different organs in VL experimental models. In contrast, the high sensitivity, accuracy, and reproducibility of real-time qPCR have led some researchers to use this technique to quantify tissue parasitism in mice and dogs naturally infected by *L. infantum* [23], [24].

The hamster is considered an important experimental model in VL due to its susceptibility to infection by different species of *Leishmania* and the development of clinical signs and pathological changes similar to those observed in human and canine disease. Thus, the present work focused on quantifying parasitism in the liver and spleen, as well as the humoral immune response evaluation and the analysis of clinical pathological pictures and the survival ratio of hamsters experimentally infected by different routes (intradermal, intraperitoneal, and intracardiac) and with different strains of *L. infantum* (MHOM/BR/74/PP75 and Wild), while also comparing two different methodologies (LDU index and qPCR).

## Methods

### Ethics Statement

Details of the project were submitted and approved by the Ethical Committee on Animal Research of the Universidade Federal de Ouro Preto (approval ID number 2009/09). All procedures were carried out in compliance with current Brazilian Regulations relating to Experimental Biology and Medicine as described in the guidelines issued by the Colégio Brasileiro de Experimentação Animal (COBEA, 2006). Experimental animals were maintained in the central animal facility at the Universidade Federal de Ouro Preto (UFOP), Minas Gerais, Brazil.

### PCR-RFLP

The PCR-RFLP assay was performed to characterize the Wild-type strain used in this work, suggested by Coura-Vital et al. [25]; the described primers used to amplify the conserved region of the *Leishmania* kDNA minicircle were as follows: forward: 59-GGG (G/T)AG GGG CGT TCT (G/C) CG AA-39; reverse: 59-(G/C)(G/C)(G/C) (A/T)CT AT(A/T) TTA CAC CAA CCC C-39 [26]. The reaction mixture consisted of 16 buffer [10 mM Tris-HCl, 50 mM KCl (pH 8.8)], 1.5 mM MgCl_2_, 2.0 mM dNTP, 1.0 pmol of each primer, 0.76 U of *Taq* polymerase (Sinapse, São Paulo, SP, Brazil), 2.5 µL DNA and Milli Q water to a final volume of 12.5 µL/well (MicroAmpH Fast Optical 96-Wells, Applied Biosystems, Foster City, CA, USA). PCR reactions were performed in a 96-well Verit Thermal Cycler (Applied Biosystems) using the following program: initial denaturation at 94°C for 1 min, followed by 40 cycles of 30 s at 93°C, 1 min at 64°C and 1 min at 72°C, with a final extension at 72°C for 7 min. PCR amplicons (5 µL) were digested for 3 h at 37°C in 1 U of *Hae III* (Invitrogen, Carlsbad, CA, USA) in buffer [10 mM Tris-HCl, 10 mM MgCl_2_ (pH 7.5)] and Milli Q water to a final volume of 15.0 µL/well (MicroAmpH Fast Optical 96-Well, Applied Biosystems) [27]. Restriction fragments, together with a 25 bp DNA ladder (Invitrogen), were electrophoresed in 10% polyacrylamide gels at 40 mA in 89 mM Tris base (pH 8.0), 89 mM boric acid and 2 mM EDTA. Bands were detected by silver staining, and the pattern of Wild strain was compared with those obtained using DNA from *L. (L.) amazonensis* (MHOM/BR/1973/M2269), *L*. (*V.*) *braziliensis* (MHOM/BR/1975/M2903) and *L*. (*L*.) *infantum* (WHO/MHOM/BR/74/PP75).

### Animals

One-month-old male Syrian golden hamsters (*Mesocricetus auratus*), weighing approximately 50–80 g, were obtained from the central animal facility at the Universidade Federal de Ouro Preto (UFOP). The animals were kept in appropriate cages with water and food *ad libitum* throughout the experiment time.

### *Leishmania* Parasites and Experimental Infection

**Figure 1:**
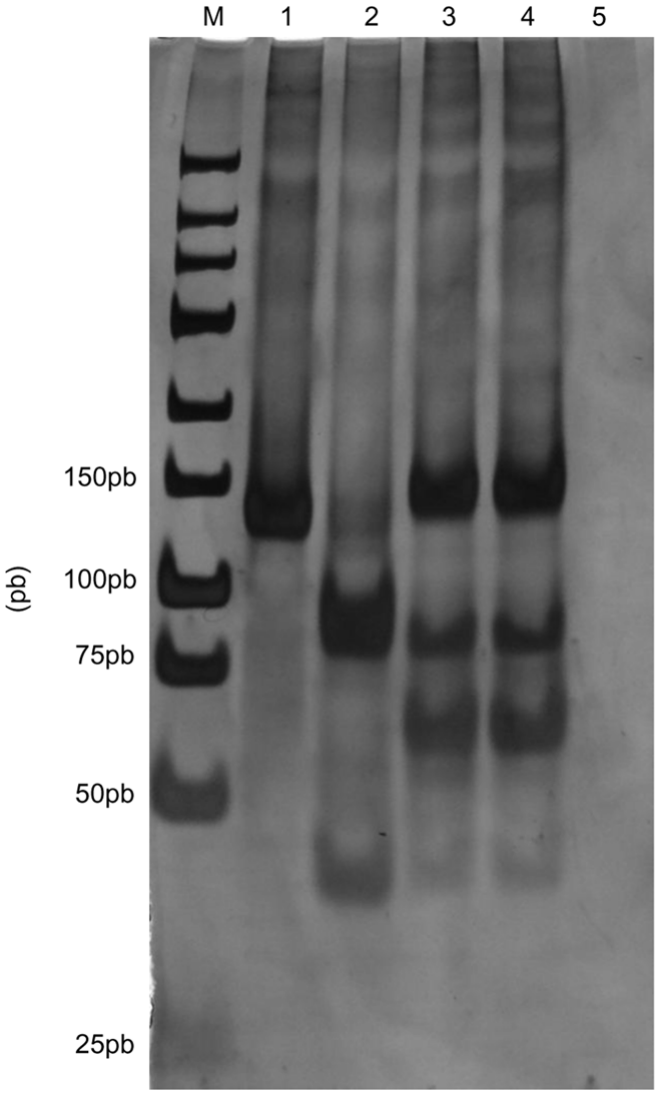
Gel electrophoresis of PCR products from different DNA samples. The PCR products were digested with *Hae III* restriction enzyme, and the digested samples were separated by electrophoresis in a 10% agarose gel to produce DNA fragments. Lane M: 25-base pair ladder; lane 1: *L. amazonensis* (MHOM/BR/1973/M2269); lane 2: *L. braziliensis* (MHOM/BR/1975/M2903); lane 3: *L. infantum* (WHO/MHOM/BR/74/PP75); lane 4: *L. infantum* (wild strain) and lane 5: negative control.

Two strains of *L. infantum* were used in this work: a reference strain of the World Health Organization, PP75 (WHO/MHOM/BR/74/PP75) and a wild-type strain (Wild), isolated from a symptomatic naturally infected dog provided by the Center of Zoonosis Control (CCZ), Governador Valadares, Minas Gerais, Brazil. RFLP-PCR analysis of both *Leishmania* strains used in this study confirmed their species as *L. infantum* (Figure 1). Parasites were grown in LIT medium (liver infusion tryptose) and NNN (McNeal, Novy & Nicolle). To obtain the promastigotes in the stationary growth phase, culture has been expanded from an initial inoculum of 10 ml of LIT medium containing between 10^7^–10^8^ promastigotes/ml in logarithmic growth. The promastigotes were added to 40 mL of culture medium NNN/LIT and are then packaged in a temperature of 23°C ±1°C. Growth curves for each strain were performed to identify the best days to recover metacyclic promastigotes in the stationary phase of growth. For both strains the seventh day was defined as an ideal growth. After seven days of the initial inoculum was held the first spike after the feasibility and noted the absence of contaminants with the aid of optical microscope, and distributed in five new Erlenmeyer flasks under the same conditions of initial inoculum (10 ml of culture in 40 mL of LIT medium). At the end of seven days, 250 ml of culture were obtained at concentrations of 10^8^ promastigotes/mL. At the end of 7 days, the culture was removed and transferred to sterile polypropylene tubes (Falcon®, Becton Dickinson, USA) and subjected to centrifugation at 900×*g* for 15 min at room temperature. The parasites were washed three times with saline at 900×*g* for 10 min, resuspended in saline, and adjusted to 1×10^7^ promastigotes per inoculum.

### Experimental Groups, Follow-up and Collection of Samples

The hamsters were subdivided into two major experimental groups according to the strain used: 120 animals were infected with the reference *L. infantum* strain (WHO/MHOM/BR/74/PP75) and another 120 animals were infected with the Wild strain. For each strain, three different routes of inoculation were used: intradermal (ID, *n* = 40), intraperitoneal (IP, *n* = 40), and intracardiac (IC, *n* = 40). The inoculum of 1×10^7^ promastigotes of *L. infantum* in the stationary growth phase culture was concentrated in a final volume in accordance with each of the three routes used. The intradermal route was made in the right ear of each animal in an amount of 20 µl and the intraperitoneal inoculum was performed in a volume of 500 µl. Finally, the intracardiac inoculum was performed in a final volume of 200 µl in animals anesthetized with 2.5% sodium pentobarbital at a dose of 50 mg/kg.

Uninfected animals were used as a control group (C, *n* = 80). The animals were followed for 9 months post-infection for survival analysis. For each time point of evaluation (1, 3, 6, and 9 months), 10 animals per group were anesthetized and blood samples were collected and sera were separated by centrifugation and collected for estimation of serum antibodies. Subsequently the hamsters were euthanized and liver and spleen were collected aseptically to quantify the parasite burden.

### Humoral Immune Response: Total IgG Anti-*Leishmania Infantum*

Serum was used to determine the anti-*Leishmania* IgG titres by the determination of antibodies against a soluble *L*. *infantum* (MHOM/BR/1972/BH46) promastigotes antigen (SLA) according to the conventional enzyme-linked immunosorbent assay (ELISA) [28]. The protein concentration was quantified by the Lowry method, adjusted to 1 mg/mL and samples were stored at −70°C. Ninety-six well microplates (MaxiSorp, Nalge Nunc) were coated with SLA at a concentration of 2 µg/well, left overnight at 4–8°C and then washed. Later it was performed the block nonspecific applying 100µL/well of PBS solution with 5% FBS (fetal bovine serum) and then washed. Serum samples were added to the wells at a dilution of 1∶80 and incubated overnight at 4–8°C. After incubation, the plate was washed and added the goat anti-hamster IgG (1∶3000, Goat Anti-Hamster - Caltag Laboratories). Wells were washed and then the substrate and chromogen (*O*-phenylenediamine, Sigma–Aldrich) were added. The absorbance was read on an automatic ELISA microplate reader (EL 800G PC, Bio-Tek) at 490 nm. The concentrations of conjugate were determined by a block titration method with positive and negative standard sera. The cut-off value was established as the mean absorbance value +2 SD from 20 serum samples from uninfected controls hamsters.

### Parasitological Analysis-parasite Load Index

Parasitological diagnosis in tissue smears (spleen and liver) was performed after necropsy of the animals. Tissue samples were collected and imprints were performed on two microscopic slides and after air-drying. Samples were fixed in methanol, stained by Giemsa, and examined under optical microscopy to identify *Leishmania* amastigote forms. The parasite density evaluation was performed on spleen and liver imprints, and the results were expressed as LDU index values, according to Stauber [19]. These values correspond to the number of *Leishmania* amastigotes per 1000 nucleated cells multiplied by the organ weight (g).

### DNA Extraction and Quantitative Real-time PCR

After the animals were euthanized, the abdominal cavity was opened and the liver and spleen were removed. Good laboratory practice was used in order to avoid DNA cross-contamination, and negative controls were included during all the DNA extraction procedures and qPCR steps. Total genomic DNA was extracted from approximately 20 mg of tissue (spleen and liver). For spleen samples, extraction with Wizard™ Genomic DNA Purification Kit (Promega®, Madison, WI, USA) was used following manufacturer’s recommendations. To obtain the DNA of the liver samples the CTAB method was used as follow: 250 µL of lysis buffer (5 mM Tris pH 7.5, 1 mM EDTA, n-lauryl sarcosine 1%) was added to the tubes containing the fragments of liver (20 mg). The mixture was incubated on dry bath at 37°C for 1 h. Afterward, 20 µL of proteinase K (20 mg/mL) was added with mixing, and the suspensions were incubated for 18 hours at 55°C. Thereafter, 100 µL of 5 M NaCl was added, incubation continued for 10 min at 65°C, and 50 µL of 10% CTAB solution (v/v) was subsequently added, followed by 20 min of incubation at 65°C. Next, 400 µL of chloroform (Sigma, St. Louis, MO, USA) was added under agitation by vortexing. The samples were centrifuged at 12,000×*g* for 10 min. The aqueous phase was transferred to a new tube and 400 µL of isopropanol (Merck®, Darmstadt, Germany) was added, and the mixture incubated at −20°C for 1 hour. After precipitation, the DNA was centrifuged for 10 min at 12,000×*g*, the supernatant was discarded, and the pellet was washed with 70% cold ethanol (Merck®, Darmstadt, Germany). Following centrifugation for 5 min at 12,000×*g*, the supernatant was discarded and the DNA precipitate was homogenized in 50 µL of nuclease-free water. The concentration of DNA obtained from tissues was determined with a spectrophotometer (NanoVue Plus, GE Healthcare Products, Piscataway, NJ, USA).

### Quantitative Real-time PCR

In order to quantify parasite burdens, we used primers described by Bretagne et al. [23] that amplified a 90-bp fragment of a single-copy of the gene of DNA polymerase of *L. infantum* (GenBank accession number AF009147). PCR was carried out in a final volume of 25 µL containing 200 nM forward and reverse primers, 1×SYBER GREEN reaction master mix (Applied Biosystems, USA), and 5 µL of template DNA. PCR conditions were as follows: an initial denaturation step at 95°C for 10 min followed by 40 cycles of denaturation at 95°C for 15 s and annealing/extension at 60°C for 1 min. Standard curves were prepared for each run using known quantities of pGEM® T plasmids (Promega, USA) containing inserts of interest. In order to verify the integrity of the samples, the same procedure was carried out for the TNF-α gene (86 bp fragment GenBank accession number AF046215). Reactions were processed and analyzed in an ABI Prism 7500-Sequence Detection System (Applied Biosystems, USA). The results were expressed as the number of amastigotes in 20 mg of tissue multiplied by the total weight of the organ (spleen and liver).

### Statistical Analysis

Statistical analysis was performed with the aid of Prism 5.0 software package (Prism Software, Irvine, CA, USA). Normality of the data was established using the Kolmogorov-Smirnoff test. One-way analysis of variance (ANOVA) and Tukey post-tests were used to investigate differences between groups. Considering the nonparametric nature of ELISA data, Kruskal-Wallis tests were used to investigate differences between the four groups, followed by Dunn’s test for pairwise comparisons. Spearman’s rank correlation was also computed to investigate associations between IgG levels with months post infection and experimental groups (ID, IP and IC). The results were considered statistically significant when *p*<0.05.

## Results

### The PCR-RFLP Pattern Identified Wild-type Strain as *L. infantum*

The PCR-RFLP pattern used in this work identified Wild-type strain as a pattern of *L. infantum*. The species were clearly differentiated by single digestion with *Hae III* restriction enzyme (Figure 1).

### Infection with the Wild Strain by the IC Route Resulted in Higher Mortality

**Figure 2:**
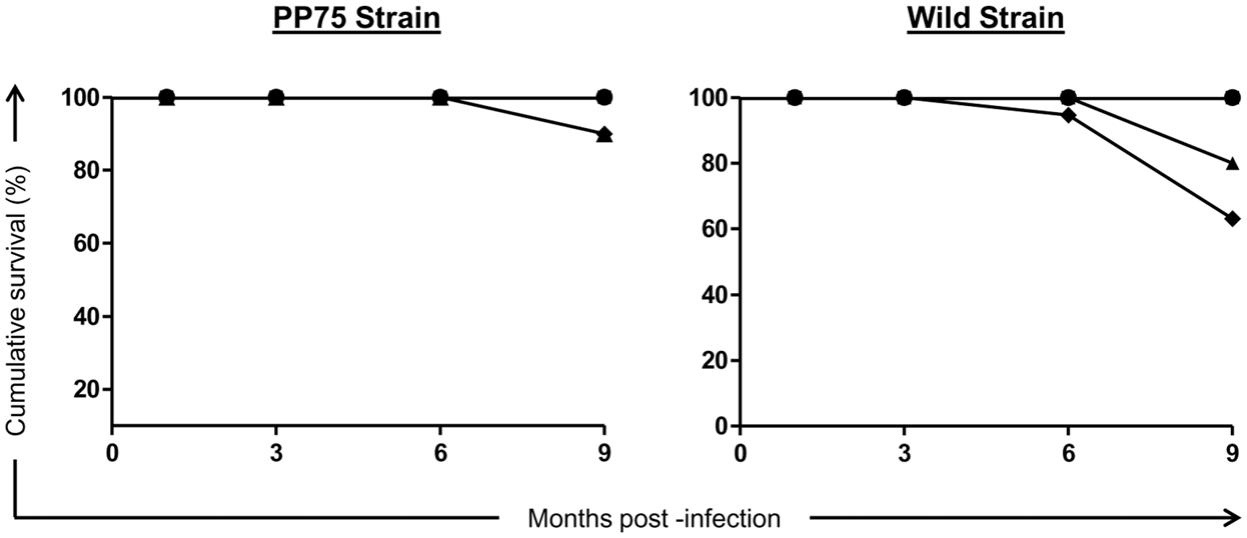
Cumulative survival in hamsters experimentally infected with two strains of *L. infantum* (PP75 and Wild) by different routes of inoculation assessed at 1, 3, 6, and 9 months after infection. Control group (C; black circle) and experimentally infected animals with two strains of *L. infantum* (PP75 and Wild) by different routes of inoculation: intradermal (ID; black square); intraperitoneal (IP; black triangle), and intracardiac (IC; black diamond).

The survival rate was 100% for the hamsters infected with both PP75 and Wild strains by the ID route. However, the hamsters infected with the PP75 strain by the IC and IP routes had a survival rate of 90%. Animals infected with the Wild strain via the IP and IC routes had survival rates of 80% and 60%, respectively (Figure 2).

In the evaluation of clinical signs at 9 months after infection, the animals inoculated with the PP75 strain via IC presented splenomegaly (77%), weight loss (22%) and hepatomegaly (33%). In contrast, in animals inoculated with Wild strain, it was observed that 87% of hamsters infected by IP route showed splenomegaly and 100% of infected animals through IC route showed ascites, splenomegaly and weight loss/cachexia. Interestingly, at 9 months after infection with the Wild strain using the IC route it was also possible to observe mucocutaneous lesions accompanied by ulcers found at the muzzle and swelling in the legs of 100% of the animals.

### Evaluation of Humoral Immune Response Post-infection Detected High IgG Levels as Marker of Clinical Progression of VL in Hamster

**Figure 3:**
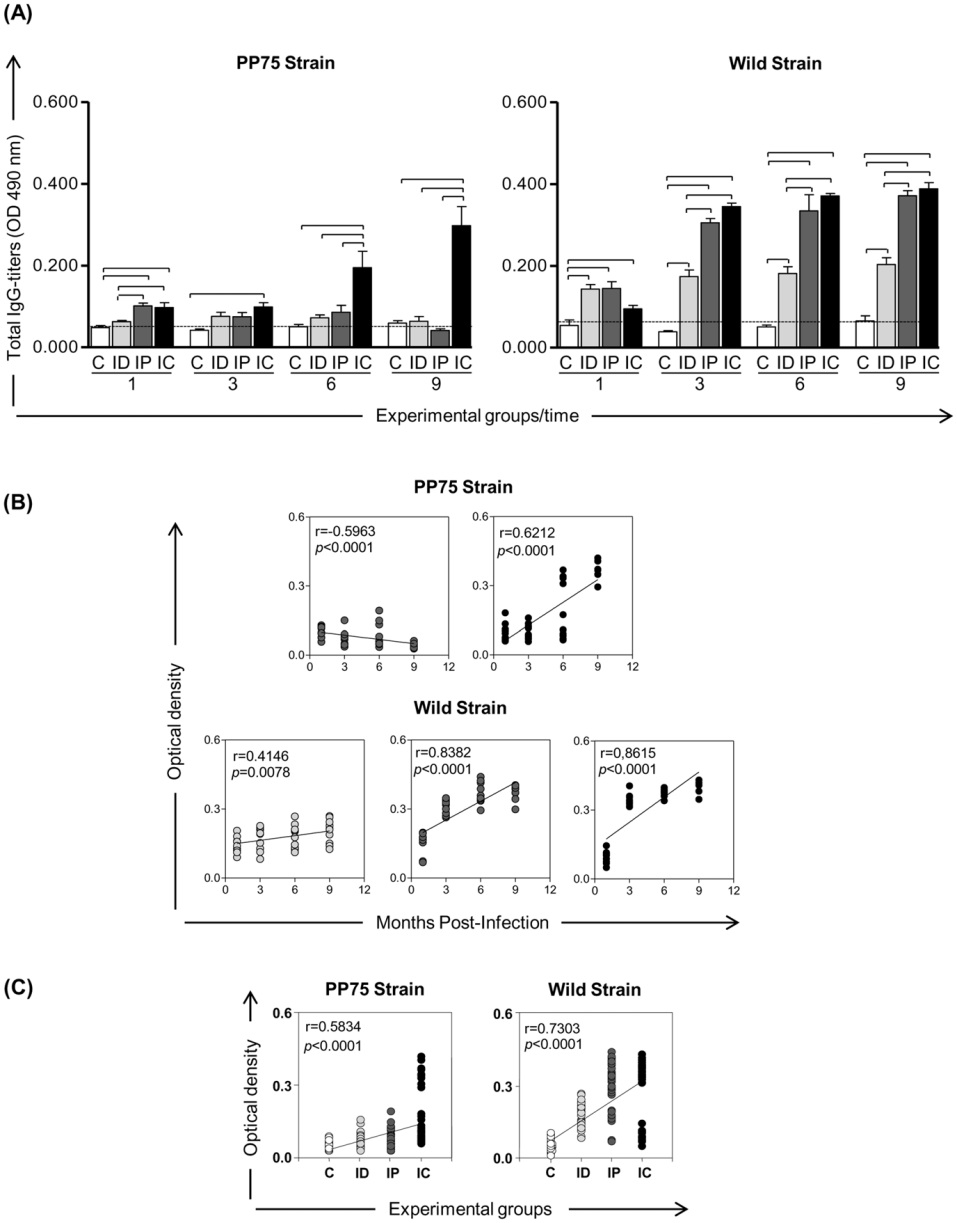
Evaluation of humoral immune response in hamsters experimentally infected with two strains of *L. infantum* (PP75 and Wild) by different routes of inoculation. (A) Means of the *Leishmania*-specific IgG levels. (B) Correlation between IgG levels and months post-infection. (C) Correlation between IgG levels and experimental groups. IgG levels were determined by ELISA in sera of uninfected hamsters as a control group (C; white) and hamsters infected with *L. infantum* from intradermal (ID; light gray), intraperitoneal (IP; dark gray) and intracardiac (IC; black) routes. The results are expressed as mean ± standard deviation. The cut-off is represented by the line. Significant differences (*p*<0.05) between infection with the different routes of inoculation are represented by the connected lines. Spearman’s correlation indexes (*r* and *P*-values) are shown on the graphs and the connecting lines illustrate positive and negative correlation indexes.

The reactivity of seric anti-*Leishmania* immunoglobulin IgG from challenged hamsters is shown in Figure 3A. Hamsters infected with PP75 strain showed significant increase on IgG levels only in IC group at all time points evaluated (1, 3, 6 and 9 months). On the other hand, all hamsters infected with Wild strain in the three experimental groups (ID, IP and IC), developed significant humoral response against SLA at all time points evaluated (Figure 3A).

A detailed analysis of the correlations between IgG levels, months post-infection and experimental groups are shown in Figure 3B and 3C. Correlations analysis in hamsters infected with PP75 strain revealed that there was a negative correlation between IgG levels and months post infection in IP group and positive correlation in the IC group. Otherwise, in hamsters infected with Wild strain all groups (ID, IP and IC) showed positive correlations between IgG levels and months post infection (Figure 3B). It was also observed positive correlations between the IgG levels and experimental groups (ID, IP and IC) in both, PP75 and Wild strains (Figure 3C).

### The IC Route Induced Higher Parasitism in the Liver and Spleen

**Figure 4:**
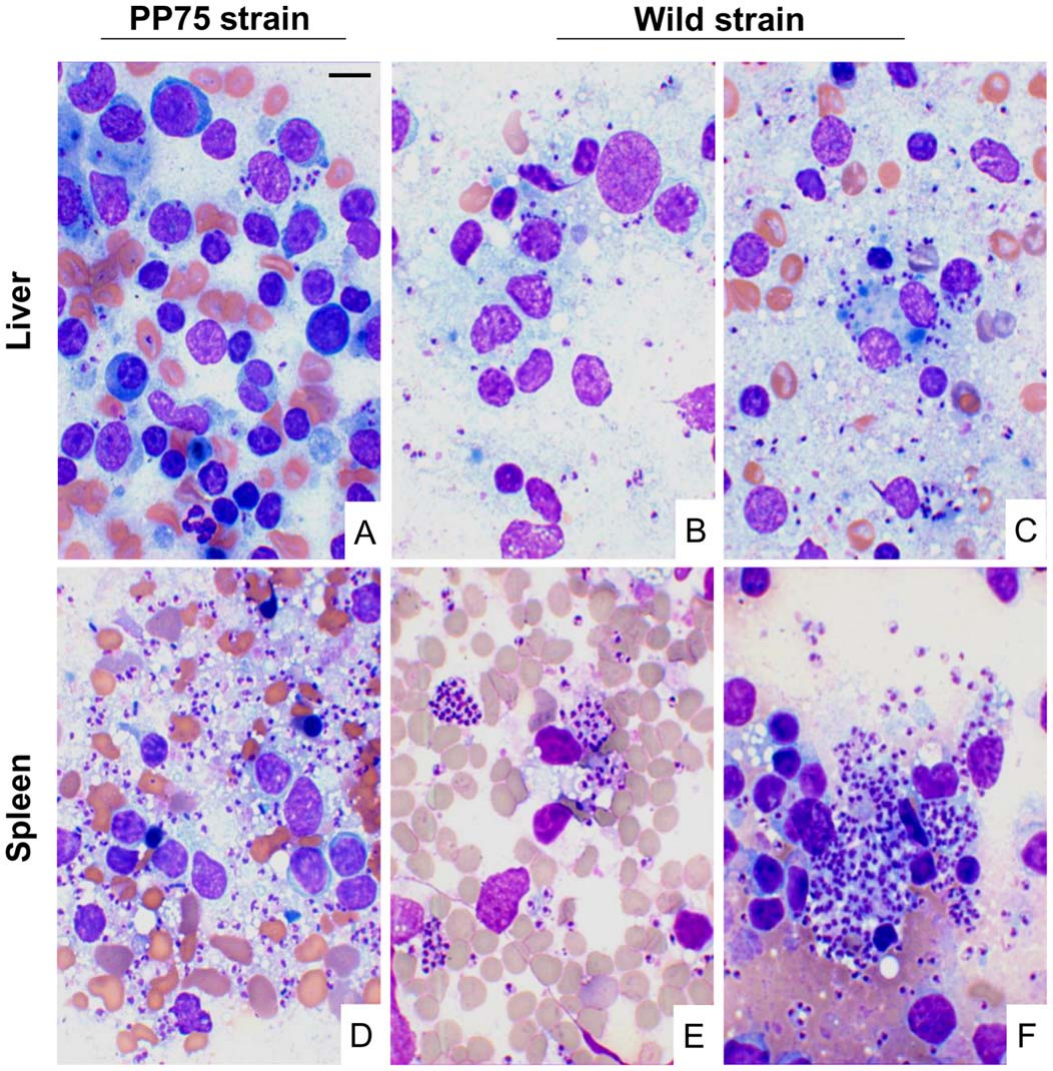
Photomicrographs of liver and spleen smears in hamsters experimentally infected with two strains of *L. infantum* (PP75 or Wild) at 9 months after infection. Presence of few amastigotes in animals infected with PP75 strain via the IC route (A) and infected with the Wild strain by the IP route (B–E) and IC (C). Intense splenic parasitism in animals infected with either PP75 (D) or Wild (F) by the IC route. Giemsa. Bar = 50 µm.

**Figure 5:**
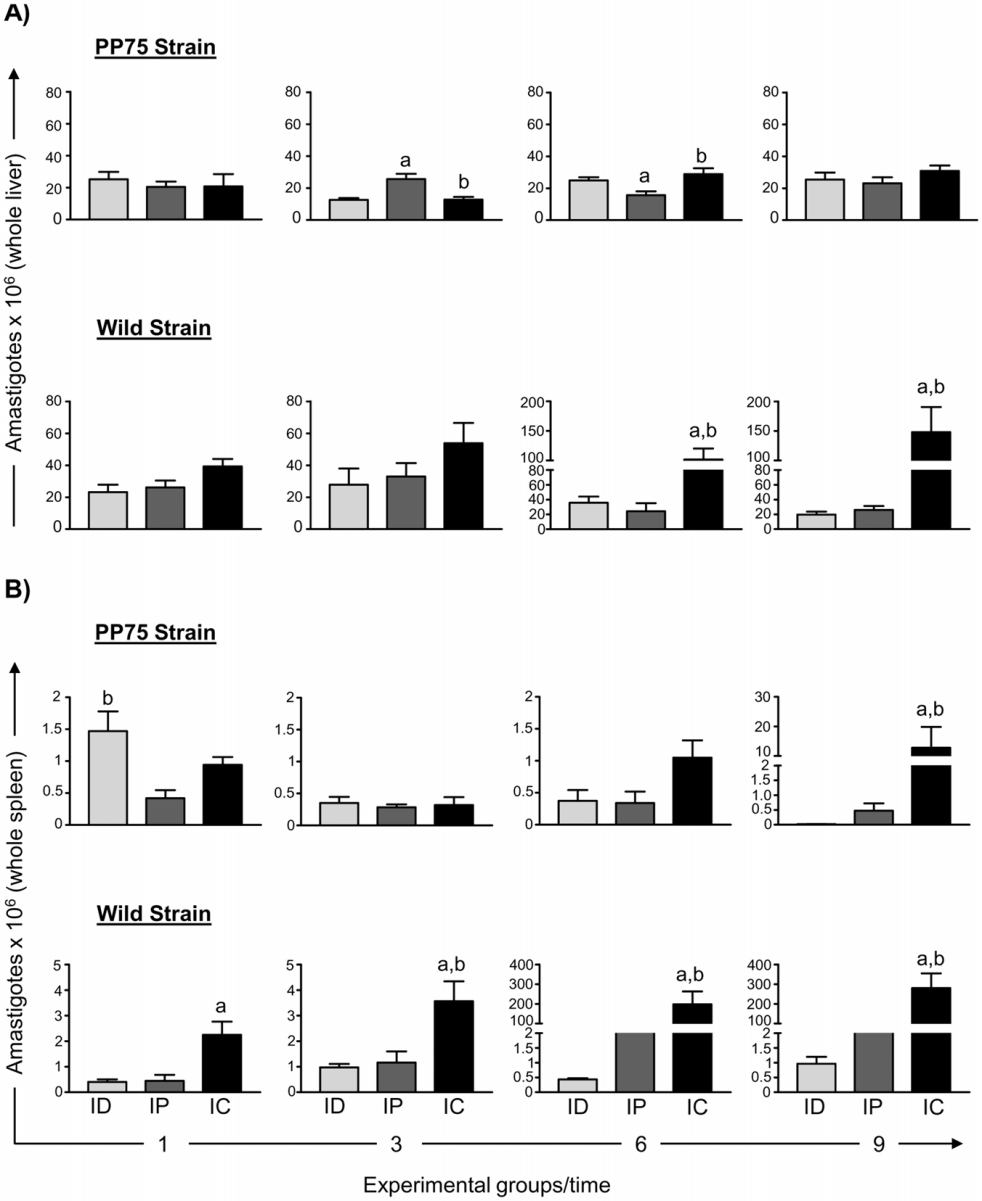
Parasite burden in hamsters experimentally infected with two strains of *L. infantum* (PP75 and Wild) by different routes of inoculation. Number of amastigotes deduced from the qPCR data by the total weight of the liver (A) and spleen (B) in hamsters experimentally infected with two strains of *L. infantum* (PP75 or Wild) by different routes of inoculation: intradermal (ID, *n* = 80; light gray), intraperitoneal (IP, *n* = 80; dark gray), and intracardiac (IC, *n* = 80; black) after 1, 3, 6, and 9 months of infection. The results are expressed as mean ± standard deviation. Significant differences (*p*<0.05) between infection with the different routes of inoculation are represented by the letters “a” and “b” related to the group ID and IP, respectively.

In order to evaluate the evolution of the infection in hamsters experimentally infected with different *L. infantum* strains (Wild and PP75) by distinct routes (ID, IP, and IC), quantification of the parasite load was performed on liver and spleen samples, and the LDU index was determined. We also employed real-time qPCR techniques to quantify the DNA from *L. infantum*. These results are shown in Table 1, Figures 4 and 5, respectively.

**Table 1:** Frequency of positivity and parasite density by LDU index values in the liver and spleen of hamsters experimentally inoculated with PP75 or Wild *L. infantum* strains.

		Strain/months after infection							
Groups		PP75				Wild			
		1	3	6	9	1	3	6	9
**ID**									
	**Liver**	–	–	–	–	–	–	–	–
	**Spleen**	–	–	–	–	–	–	–	–
**IP**									
	**Liver**	–	–	–	–	–	–	6/10 (60%)	7/8 (87%)
								238.2±48^a^	220.2±337 ^a^
	**Spleen**	–	–	–	–	–	2/10 (20%)	9/10 (90%)	8/8 (100%)
							0.43±0.08	6.08±11.08^a^	147±399^a^
**IC**									
	**Liver**	–	–	4/10 (40%)	4/9 (44%)	8/10 (80%)	9/10 (90%)	10/10 (100%)	6/6 (100%)
				34.4±42.7^a,b^	218.2±281.1^a,b^	58.81±59.81^a,b^	168.8±163.5^a,b^	1325±1312^a^	3425±2634^a,b^
	**Spleen**	–	–	4/10 (40%)	7/10 (70%)	7/10 (70%)	10/10 (100%)	10/10 (100%)	6/6 (100%)
				12.13±10.3^a,b^	456.7±103^a,b^	1.61±1.13^a,b^	5.75±4.48^a,b^	827±928^a,b^	2381±19.01^a,b^

Significant differences (*p*<0.05) between infection with the different routes of inoculation are represented by “a” and “b” related to the ID and IP groups, respectively.

ID =  intradermal route; IP =  intraperitoneal route; IC =  intracardiac route.

Data obtained by LDU analysis of livers from animals infected with the PP75 strain by IC route showed higher liver parasitism at 6 and 9 months after infection (40% and 44%, respectively) (Table 1 and Figure 4). Amastigotes were not observed at 6 and 9 months in the liver of hamsters inoculated with PP75 strain by the IP and ID routes (Table 1). Moreover, in animals infected with the Wild strain by the IC route, a high liver LDU index was found compared with the ID and IP groups, with 100% positivity at 6 and 9 months after infection. However, in hamsters inoculated via the IP route, liver parasitism started at 6 months with 60% of the animals positive, followed by 87% at 9 months after infection.

During the follow-up of the animals infected with the PP75 strain, no parasitism was detected by optical microscopy evaluation of the spleen of animals inoculated by the ID and IP routes (Table 1). However, 40% and 70% of the animals inoculated with this same strain via the IC route presented spleen parasitism at 6 and 9 months after infection, respectively. In contrast, 20%, 90%, and 100% of the animals infected with Wild strain by the IP route exhibited spleen parasitism at 3, 6, and 9 months, respectively. When animals were inoculated by the IC route, it was possible to detect parasitism at 1, 3, 6 and 9 month after infection in 70%, 100%, 100%, and 100% of the hamsters, respectively (Table 1 and Figure 4).

### qPCR Showed Higher Sensitivity for Detecting a Low Parasitic Burden

The results obtained by qPCR are displayed calculating the DNA load in 20 mg of tissue multiplied by the total weight of the organ (Figure 5A and B).

The results obtained by Real Time-PCR demonstrated higher liver parasitism at 3 months after infection in the animals infected with the PP75 strain by the IP route compared with the ID and IC groups. Furthermore, at 6 months post infection, a reduction in the liver parasitic load occurred in the IP group compared with the ID and IC groups. We also observed that hamsters infected with the Wild strain via the IC route presented a higher DNA load in the liver at 6 and 9 months in comparison with the ID and IP groups (Figure 5A).

Additionally, we observed higher spleen parasitism in animals inoculated with the PP75 strain by the ID route than the IP route 1 month after infection. The analysis of parasite burden in the spleen showed that animals infected with the PP75 strain at 9 months after infection presented a higher parasite load if infected by the IC route compared with the hamsters inoculated by the ID and IP routes (Figure 5B).

Animals infected with the Wild strain via the IC route presented a higher parasitic load in comparison with the ID group in the first month after infection (Figure 5B). Further, at 3, 6, and 9 months, the IC group had higher spleen parasitism when compared with both the ID and IP groups (Figure 5B).

## Discussion

In the present study we evaluated for the first time mortality, humoral response and parasitism in hamsters experimentally infected via different routes (ID, IP, and IC) and with different strains of *L. infantum* (MHOM/BR/74/PP75 and Wild) that had distinct degrees of virulence and pathogenicity. Animals were followed for 9 months after infection.

In the present study, we demonstrated that increased levels of IgG detected in animals inoculated with PP75 strain were observed later, with higher peaks at 6 and 9 months after experimental infection in the IC group. However, in animals infected with the Wild strain the IgG levels were elevated in all infected groups during all the time evaluated. These data demonstrate that the Wild strain was able to induce higher levels of IgG in the earliest time regardless the route of inoculum, especially in more advanced stages of disease where animals showed clinical VL symptoms (ascites, splenomegaly and weight loss/cachexia). Our results are consistent with Requena et al. [7] who associated the strong humoral response in hamsters experimentally infected with *L. infantum* to the presence of clinical signs and elevated parasite tissue density. Other studies have shown a relationship between parasite load and high antibody titers in hamsters experimentally infected with different forms and species of *Leishmania* [6], [21], [22], [29]. Thus, similarly to human and canine LV, the evaluation of anti-*Leishmania* antibody is an important tool in monitoring the establishment of infection in the hamster model.

Besides these results, we used two techniques to quantify parasitism in different visceral target organs (spleen and liver): LDU index and qPCR. Quantifying parasitism is fundamentally important to comprehend the clinical manifestations and pathological changes that occur during the natural history of experimental VL. We evaluated the impact of parasite load in two compartments (spleen and liver) after infection by *L. infantum* strains. The development of molecular approaches to quantify the parasite load in the spleen and liver from experimentally infected hamsters is extremely relevant because this animal model has been used to evaluated potential drugs and candidate vaccines against VL [30], [31], [32], [33], [34], [35], [36], [37], [38]. The LDU index, as described by Stauber [19], has the disadvantage of low sensitivity for detecting amastigotes by optical microscopy. Moreover, reproducibility in detecting amastigotes requires a trained technician because different results can be generated by different microscopists. Finally, a further disadvantage is that the technique relies on the dispersion of the parasites in the imprints stained with Giemsa, which are distributed in the sample in a nonuniform way [23]. The real-time PCR technique has been used by several authors to diagnose or monitor the evolution of VL in mice [17], [23], [39], dogs [24], [40], and humans [40], [41], [42], [43], [44], [45] through quantification of parasite load, and the method may replace other techniques due to its high sensitivity, accuracy, and reproducibility [23], [24], [46]. Nevertheless, real-time PCR for quantifying parasitism in visceral organs from hamsters experimentally infected with *L. infantum* had not been performed.

We observed that in animals infected with both strains, the parasite load evolved with the progression of the infection. In addition, parasitism in the liver and spleen of animals infected with the Wild strain was higher compared with those infected with the PP75 strain. We also demonstrated that the spleen was the organ most intensely parasitized in both strains. According to Ott et al. [47], the parasitism measured by the LDU index in the spleen is more consistent if compared with the liver, probably because the difference in size and weight between them. Studies conducted by Binhazim et al. [48] using hamster experimentally infected with *L. infantum* via the IC route, showed higher parasitism in the liver compared with the spleen. Given the number of amastigotes per 1000 nuclei cells but without consideration of the organ weight, the spleen appeared to be more parasitized than the liver. Higher splenic parasitism has been shown by many other researchers in studies that used hamsters experimentally infected by *L. infantum* and/or *L. donovani* with different infective forms (amastigote and promastigote) and routes of inoculation [6], [7], [19], [21], [22], [47]. The discrepancy in results presented by others authors can be explained because of the use of limiting dilution for assessing parasitism in spleen and liver without including organ weight in the calculation of parasite load.

In our study, the LDU index showed low sensitivity when compared with qPCR, detecting parasites only in the IP and IC groups, while qPCR was able to detect DNA load of the *L. infantum* in animals inoculated by all routes at all study time points (1, 3, 6, and 9 months) after infection. Our results demonstrate that the use of the LDU index may make detection of amastigotes by optical microscopy difficult in experimentally infected hamsters with asymptomatic disease; these animals displayed low parasite load in visceral organs, as shown in animals inoculated by the ID route with either strain. With the IP route, a long period after infection (6 and 9 months) was needed prior to being able to visualize and quantify the amastigotes by optical microscopy in the spleen and liver. According to Ott et al. [47] after inoculum into the peritoneum, a large number of the parasites died, thus prejudicing the establishment of the infection in the spleen and liver and consequently enlarging the prepatency period.

The results demonstrated that the IC route induced a higher parasite density that was able to generate progressive and fatal disease in these animals. Hamsters inoculated by the IC route usually experience a progressive infection with higher values in the parasite load and display many clinical signs [19]. These findings corroborate those obtained in our work, since significant differences were demonstrated in the LDU index obtained from the groups infected with the Wild strain by the IP and IC routes in both organs evaluated.

These data emphasize that the evolution of experimental infection depends on several factors, including the virulence of the parasite, the route of inoculation, the organ evaluated, and even the time after infection [5], [8], [47], [49], [50], [51].

Considering that more virulent and pathogenic the strain, as the Wild strain used in this work, more aggressively the infection progresses as a function of the route of inoculation and organs evaluated. The variation found in the percentages and numbers of the parasites in the spleen and liver in different experimental groups is probably related to the immune response of the animals and to the fact that the parasites do not distribute themselves uniformly in organs.

The literature is sparse about evaluations of qPCR for quantifying the parasite density (DNA load) in different organs in a hamster model infected by *L. infantum*. To quantify the DNA load in the liver and spleen of experimentally infected hamsters, standard curves constructed from known concentrations of DNA from *L. infantum* were used, which is one of the best ways to determine the results obtained by qPCR [46].

In our study, qPCR was shown to be sensitive enough to detect *L. infantum* DNA in both the spleen and liver in all experimental groups (ID, IP, and IC) independent of the strain used, and it could be used to monitor the parasite load in these organs throughout the evaluation period. Compared with the LDU index, the qPCR also demonstrated a higher parasite burden in the liver and spleen in the animals infected with the Wild strain by the IC route. These results confirm those obtained by other authors that evaluated parasitism in spleen and liver by others methods [6], [7], [21], [22].

We confirmed Stauber’s [19] hypothesis that the IC route induces higher parasitism in the spleen and consequently faster and more successful infection, suggesting that this route could be the most appropriate for obtaining a strong, progressive infection with *L. infantum* in the hamster model in a short period of time. This would be useful when the objective is to test drugs in this model [22]. The IP route produced low and variable parasite loads especially with the PP75 strain. At the end of the evaluation period, we observed some parasitism peaks with relatively higher values in animals that were infected with the Wild strain, suggesting that a longer period for the establishment of the experimental infection is required if the IP route was used. Similar results were reported by Ott et al. [47] using the LDU index to enumerate parasitism in the spleen and liver from hamsters experimentally infected with *L. donovani* by the IP route.

The ID route most closely resembles natural infection, but the disease manifests slowly with this route in the hamster model. Indeed, other groups have shown in hamster and dogs models that the ID route induces asymptomatic disease and requires a long time to establish tissue parasitism during the infection [52], [53]. Although that the ID group showed a slow evolution for the VL disease after *L. infantum* infection, the parasites in the spleen and liver were able to replicate and it was possible to quantify the burden of the parasite by qPCR in both tissues independent of the strain used. In this context, we standardized and used qPCR, a technique with higher sensitivity to detect DNA load from *Leishmania* spp. in different tissues affected by VL infection. Our results showed the benefits of qPCR for quantifying parasitism in a hamster model, which is particularly important given intense use of this model in the last decade to test VL drugs and vaccines.
